# COVID-19 and Emotional Variables in a Sample of Chileans

**DOI:** 10.3389/fpsyg.2021.615268

**Published:** 2021-01-26

**Authors:** Mariela González-Tovar, Sergio Hernández-Rodríguez

**Affiliations:** ^1^Pontificia Universidad Católica de Chile, Santiago, Chile; ^2^Central University of Venezuela, Caracas, Venezuela

**Keywords:** COVID-19, emotional variables, emotional disturbances, age, affections and emotions

## Abstract

**Introduction:**

During the Coronavirus (COVID-19) pandemic, a set of daily stressors are being experienced, all this affects people’s mental health, leading them to have a set of emotional disturbances. Little is known about how people’s age can influence their emotional well-being in the face of prolonged stress generate by the pandemic.

**Objective:**

To clarify the presence of emotional aspects such as emotional expressiveness and the frequency of positive and negative affections in people with different age in times of crisis.

**Methods:**

The final sample included 297 Chileans between 22 and 68 years old (*M* = 38.51, *SD* = 13.85), recruited through an online survey with the appropriate written informed consent. The study was carried out when the pandemic was spreading in Chile.

**Results:**

The findings revealed age differences in emotional expressivity and the type of affections experienced. The expression of emotions was more affected by negative affections, the age and the gender of the people. While the avoidance of this emotional expression, by age and affections both positive and negative. Age was a significant predictor of emotional expressiveness.

**Discussion:**

Findings suggests that the associations between both variables, varied according to the age group of the people. Furthermore, this finding proposes that although older people are facing the persistent and serious threat of COVID-19, they show better emotional functioning. Which would help to better understand the interaction of both positive and negative life experiences in times of crisis.

## Introduction

Coronaviruses are a family of viruses that cause diseases ranging from the common flu to more serious illnesses (WHO, 2020a). 2019-nCoV is a new strain that has not been previously identified in humans and causes coronavirus disease (COVID-19).

COVID-19 was identified in China in December 2019, when a pneumonia of unknown cause was detected in Wuhan, China (Reynolds, 2020). On January 30, 2020, the WHO declares that the COVID-19 outbreak constitutes a Public Health Emergency of international importance. On March 11, 2020, it is declared a global pandemic, given the high spread of the virus worldwide. On June 15, 2020, internationally, there are already 216 countries reporting cases of COVID-19, with a total of 7,805,148 confirmed cases and 431,192 deaths.

The virus was confirmed to have reached Chile in March 2020 (Garrison, 2020). As of 11 June 2020, Chile has the third largest number of cases in South America and in Latin America, after Brazil and Peru. While the number of fatalities has been lower than other countries in the region, even with fewer cases declared, the number of cases and fatalities have increased constantly since May 2020 (Garrison, 2020).

COVID-19 is a global public health crisis of a scale not previously experienced in modern times (Kickbusch et al., 2020). Authorities worldwide have responded by implementing travel restrictions, lockdowns, workplace hazard controls, and facility closures. The pandemic has caused global social and economic disruption (Nicola et al., 2020). It has led to the postponement or cancelation a lot of events (Deb et al., 2020). Widespread supply shortages exacerbated by panic buying (Scipioni, 2020). Schools, universities, and colleges have been closed either on a nationwide or local basis in 177 countries (UNESCO, 2020).

Some countries were put under mandatory quarantine due to an increase of cases. Quarantine involves the restriction of movement, or separation from the rest of the population, of healthy persons who may have been exposed to the virus, with the objective of monitoring their symptoms and ensuring early detection of cases (WHO, 2020b). Quarantine is different from isolation, which is the separation of ill or infected persons from others to prevent the spread of infection or contamination (UNICEF, 2020).

This quarantine and/or isolation has had a substantive societal impact that permeates almost every facet of daily life (Gostin and Wiley, 2020; Shanafelt et al., 2020). These widespread changes represent considerable sources of stress in the population and will have deleterious effects on mental and physical health going forward (Hagger et al., 2020).

The health threat posed by the COVID-19, and concerns about its effects on family, friends, and age group, denotes a substantive source of stress itself (Hagger et al., 2020). This stress arising from the pandemic and associated lockdown measures is likely to be prolonged even after the threat of the virus has passed (Hagger et al., 2020).

The prolonged exposure to stress arising from the crisis is likely to have insidious long-term health effects including increased risk of physical (e.g., chronic disease risk) and mental (e.g., depression, anxiety disorders, post-traumatic stress disorder) (Gonzalez, 2020; Gostin et al., 2020; Hagger et al., 2020).

An increase in symptoms of stress, depression and anxiety are already being reported in several countries in relation to the COVID-19 pandemic (Li et al., 2020; Rajkumar, 2020; Wang et al., 2020; Xiao, 2020; Xiao et al., 2020). These researches indicate that the prevalence of depression is about 38%. Furthermore, it has been determined that the psychological analysis that measure the level of anxiety and emotionality shows that 71.5% of population has anxiety symptoms.

Anxiety is a normal reaction to uncertainty and things that may harm us (Grupe and Nitschke, 2013). However, too much anxiety can start to cause harm. Feeling stressed and fearful every day takes a toll on health and well-being very quickly (Asmundson and Taylor, 2020a,b).

According to stress theory and perceived risk theory (Norris et al., 2002), public health emergencies trigger more negative emotions and affect cognitive assessment as well. It is widely accepted that emotional states disturb people’s behavior but not always in the same way (Garaigordobil and Berrueco, 2007; Esnaola et al., 2008; Dave et al., 2011), examining age differences in responses to population-wide stressors may shed light on important theoretical questions about differences in emotional experience and emotion regulation (Carstensen et al., 2020).

There is substantial evidence that, on balance, older people’s daily emotional experience is more positive than younger people’s (Carstensen et al., 2011; Burr et al., 2020). However, it is unclear whether this relatively positive emotional profile reflects improved regulation of experienced emotions or the active avoidance of environments that elicit negative emotions (Carstensen et al., 2020).

Extensive theoretical work has been devoted to explaining these widely documented age associations with emotional well-being. Some theories suggest that age-related advantages reflect the avoidance of stressors (Charles et al., 2009; Carstensen et al., 2020), while others maintain that age advantages are driven by motivational shifts that direct cognitive and behavioral resources to positive and meaningful aspects of life (Carstensen et al., 2011).

The literature on it suggests that differential emotion processing is a predeterminate mode of processing that emerges across adulthood and involves minimal cognitive effort in directing attention to positive elements of life (Allard et al., 2010).

In one research, Charles et al. (2009) detected that older people experienced less affective reactivity than younger people when they were able to avoid stressful or tense interactions. In the same way, Birditt et al. (2011) found that older people tend to use avoidant coping strategies in response to negative experiences, such as ignoring the problem or doing nothing, and less likely to use direct-negative strategies such as arguing or yelling than younger people.

An important unresolved issue is whether age-related gains in emotional experience rest principally on avoiding stressors or whether other factors; for example, if some people oldest or younger are more or less emotional, or if this effect depending of other factors (biopsychosocial) (Poon and Cohen-Mansfield, 2011).

The analysis of these aspects would be of fundamental importance at the time of planning treatment to manage the COVID-19 pandemic, its restrictions and consequences. This is because they are positively associated with self-esteem, well-being, satisfaction with life, social contact, and negatively with social anhedonia (Gross and John, 2003). Furthermore, their deficits have been found to be associated with depression (Sloan et al., 2001), and post-traumatic stress disorder (Tull et al., 2007). If these aspects are ignored, these can hinder and interfere with the success of the treatment, which certainly creates a need of conducting researches with different methodologies useful to give an account of the presence and relations among these different variables.

On the other hand, research over the past two decades shows that culture and sociodemographic characteristics (e.g., gender, race, education) have an important influence in experiencing, expressing and labeling emotions (Hofstede, 1983; Matsumoto, 1993). Emotional expressiveness may vary by several sociodemographic characteristics. Different studies on culture (Pennebaker et al., 1996; Basabe et al., 2000) have found gender differences in the frequency of emotional expressiveness. It is very common to find that women tend to report more emotional expressiveness and negative affections than men (Almeida and Kessler, 1998). However, this would depend on the social and/or race differences of the people, since it has been shown that there seems to be a normative system of rules for emotional display according to the upbringing and education of each person.

Given the importance of emotional aspects in the life of the individual, the present study categorized as relevant, to clarify the presence of emotional aspects such as emotional expressiveness and the frequency of positive and negative affections in people with different age in the time of COVID-19, considering gender as a covariate, to control its effect on the emotional response of the participants.

This investigation addressed the following questions:

(1) Are there age differences in the types of reactions reported in the time of COVID-19?
(2) Can age together with the frequency of positive or negative affections predict emotional expression?

## Materials and Methods

### Design of the Study

This is cross-sectional study that involves looking at data from a population at one specific point in time.

### Participants

The data was collected between April and May 2020, the sample was composed of 297 adults recruited trough via study advertisements in the internet, e-mail, and recommendations by previous participants in other researches.

Participants were informed on the objectives and procedures of this study, and it was conducted according to the Declaration of Helsinki. All participants gave their written informed consent before being included in the recruitment phase and completed all the measures in the survey.

The sample consisted of 74 men (24.9%) and 223 women (75.1%). Age ranged from 22 to 68 years, with a mean age of 38.51 years (*SD* = 13.85). Table 1 presents the participants characteristics.

**TABLE 1:** Characteristics of the sample.

	Media	*SD*	*N*	%
**Gender**				
Feminine			223	75.10%
Masculine			74	24.90%
**Age (years old)**				
18–29	38.51	13.85	62	32%
30–39			71	22.90%
40–49			41	23.90%
50–59			28	13.80%
60–69			28	9.40%

### Instruments

#### Emotional Expressivity Scale (EES)

Emotional Expressiveness (EE) is understood as the ability of people to express their emotional states in observable behaviors (Kring et al., 1994). It was measured using the Spanish version of the Emotional Expressivity Scale (EES) made by Kring et al. (1994). It consists of 17 items intended to measure the apparent show of emotions regardless of the valence (positive or negative) and the channel (vocal, facial, gestural) used to express them. The response format is Likert-type, with a response option from 1 (totally disagree) to 4 (totally agree) and it is corrected in an inverse manner. A higher score indicates higher expression. This inventory has a Cronbach’s alpha of 0.910 (Kring et al., 1994). In the current study, the inventory has shown a Cronbach’s alpha of 0.712.

#### Structure of Affect

Affect is regarded as a psychological construct that refers to mental states involving evaluative feelings (e.g., feeling good-bad, liking-disliking a situation) (Parkinson et al., 1996). The affective experience has two dominant dimensions, namely, positive affect (PA), and negative affect (NA) (Watson et al., 1984). PA and NA have been described as two independent unipolar dimensions of affect that include all the affective states with a positive valence (joy, enthusiasm, crush, etc.) or a negative valence (anger, fear, anxiety, etc.) (Bradburn, 1969). To assess PA and NA was using the Spanish version of the Positive and Negative Affect Scale (PANAS) by Watson et al. (1988).

PANAS contains 12 items on two subscales that assess a person’s positive and negative trait affect. The response format is Likert-type, with a response option from 1 (very slightly or not at all), to 5 (extremely). The total score achieved is obtained by adding the response values assigned to each item on one and the other scale, respectively. A higher score indicates higher frequency and/or intensity of positive and negative emotions, while a lower score shows less frequency and/or intensity of the emotions experienced as positive or negative. Both the original validation (Watson et al., 1988) and the Spanish validation (Díaz et al., 2020) showed two clearly differentiated factors (PA and NA) and good psychometric properties. In the current study, the inventory has shown a Cronbach’s alpha of 0.826.

### Another Measures

#### Age Groups

Participants reported their date of birth that was categorized into two groups: 0 (below 30 years old); 1 (over 30 years old).

It was divided into these groups because since from the age of 30 a stage begins in which important changes are generated in people, the interdependent personal and work roles governed by society and the opportunities that these give each person.

### Procedure

The data was got using an online survey distributed by e-mail to the group of people who responded to the invitation or who wanted to participate in the study. Participants were informed on the objectives and procedures of this study. The confidentiality and anonymity of their data was guaranteed, and they were asked to sign the informed consent, read the instructions, and respond to the scales. Participation in the study was voluntary.

### Ethical Considerations

The study was conducted according to the Declaration of Helsinki. All participants gave their written informed consent before being included in the recruitment phase and completed all the measures in the survey.

### Data Analysis

IBM SPSS Statistics version 24 in tandem with Python programming language version 2.7, Scikit-Learn version 0.23.1, and JupyterLab version 1.0 web-based development environment were used to analyze data.

First, descriptive analysis was performed, and normal distribution of variables was confirmed with the Kolmogorov–Smirnov test. Descriptive analysis was performed, including frequencies, means, standard deviation, to examine the distribution of scores, the need for recoding and to evaluate the performance of the scales in terms of reliability.

The differences between means were tested by Independent ANOVA. To determine the association between variables, regression was calculated.

## Results

### Descriptive Statistics

Table 2 shows the descriptive statistics of the average score.

**TABLE 2:** Descriptive statistics of the variables.

	Below 30 years old				Over 30 years old			
Variables	Min	Max	M	*SD*	Min	Max	M	*SD*
Emotional expressiveness avoidance of showing emotions	0	32	12.98	8.03	0	33	14.47	8.43
Emotional expressiveness showing emotions	0	18	10.71	3.85	0	18	11.95	4.12
Positive affections	1	28	12.18	5.54	0	36	11.25	6.86
Negative affections	10	35	22.74	5.55	9	42	24.76	6.14

Emotional expressiveness descriptors: the sample has a greater tendency to avoid emotions rather than showing them, based on the scores obtained (see Table 2).

Descriptors of positive and negative affections: the sample has a greater tendency to have a higher frequency of negative states rather than positive, based on the average of the scores obtained (see Table 2).

### Comparison Analysis Between Study Variables

The analysis of variance (ANOVA) was carried out to determine if there are significant differences by age. ANOVA by age revealed a statistically significant result for showing emotions [*F*(1, 295) = 6.33, *p* = 0.012, η^2^ = 0.021]. In addition, this analysis revealed a statistically significant result for negative affections [*F*(1, 295) = 7.66, *p* = 0.006, η^2^ = 0.025] (see Table 3). The findings indicate that people over 30 years old reported more showing emotions (*M* = 11.95) than people below 30 years old (*M* = 10.71). Similarly, people over 30 years old reported more negative affections (*M* = 24.76) people below 30 years old (*M* = 22.74).

**TABLE 3:** One-way analysis on variance (ANOVA) summary table for the effects of age on emotional expressiveness and affection.

Variable age		Source	*df*	*SS*	*M*	*F*	*p*	η^2^
Emotional expressiveness	AE	Between-group	1	147.67	147.67	2.14	0.144	0.007
		Within-group	295	20,331.06	68.92			
		Total	296	20,478.73				
	SE	Between-group	1	102.70	102.70	6.33	0.012	0.021
		Within-group	295	4,787.18	16.23			
		Total	296	4,889.88				
Affections	AP	Between-group	1	57.53	57.53	1.38	0.240	0.005
		Within-group	295	12,263.57	41.57			
		Total	296	12,321.10				
	AN	Between-group	1	271.03	271.03	7.66	0.006	0.025
		Within-group	295	10,439.03	35.39			
		Total	296	10,710.06				

*AE, avoidance of showing emotions; SE, showing emotions; AP, positive affection; AN, negative affection.*

As for the covariates, ANOVA by gender revealed a statistically significant result for showing emotions [*F*(1, 295) = 4.46, *p* = 0.036, η^2^ = 0.015]. In addition, this analysis revealed a statistically significant result for negative affections [*F*(1, 295) = 5.46, *p* = 0.020, η^2^ = 0.018] (see Table 4). Women reported more showing emotions (*M* = 11.82) than men (*M* = 10.68). Meanwhile men reported more negative affections (*M* = 24.49) than women (*M* = 23.61).

**TABLE 4:** One-way analysis on variance (ANOVA) summary table for the effects of gender on emotional expressiveness and affection.

Variable gender		Source	*df*	*SS*	*M*	*F*	*p*	η^2^
Emotional expressiveness	AE	Between-group	1	9.32	9.32	1.34	0.714	0.000
		Within-group	295	20,469.41	69.39			
		Total	296	20,478.73				
	SE	Between-group	1	72.84	72.84	4.46	0.036	0.015
		Within-group	295	4,817.04	16.33			
		Total	296	4,889.88				
Affections	AP	Between-group	1	20.33	20.33	0.49	0.486	0.002
		Within-group	295	12,300.77	41.70			
		Total	296	12,321.11				
	AN	Between-group	1	194.74	194.74	5.46	0.020	0.018
		Within-group	295	10,515.32	35.64			
		Total	296	10,710.06				

*AE, avoidance of showing emotions; SE, showing emotions; AP, positive affection; AN, negative affection.*

#### Multivariate Statistics

Multivariate regression analysis showed that age, positive affection, negative affection, and gender were significantly related to showing emotions, predicting the 10.8% of total variance (adjusted *R*^2^ = 0.108, *F* = 9.60, *p* < 0.001) on scores of showing emotions. Age, negative affection, and gender emerged as predictors (*p* < 0.05). Table 5 shows the final multiple regression model.

**TABLE 5:** Regression analysis summary predicting showing emotions.

	Adjusted *R*^2^ = 0.108						
Variables	*B*	*SD*	95% CI	β	*t*	*p*	*VIF*
Age	0.903	0.48	[0.004, 1.886]	0.105	1.89	0.041	1.029
Positive affection	–0.057	0.521	[−0.126, 0.102]	−0.091	−1.63	0.104	1.019
Negative affection	0.176	0.38	[0.108, 0.258]	0.261	4.61	0.000	1.056
Gender	1.557	0.52	[0.531, 2.583]	0.166	2.99	0.004	1.020

There was an interaction between age group, the number of negative affection and gender when predicting showing emotions.

To detect multicollinearity, variance inflation factor (VIF) was calculated. As a result, the VIF between the variables was less than 10 (see Table 5). There was no collinearity among the included variables in the regression model (Wood, 1984).

A second, multivariate regression analysis showed that age, affective affection, negative affection and gender were significantly related to avoidance showing emotions, predicting the 16.50% of total variance (adjusted *R*^2^ = 0.165, *F* = 15.60, *p* < 0.001) on scores of showing emotions. Age, positive affection and negative affection emerged as predictors (*p* < 0.05). Table 6 shows the final multiple regression model.

**TABLE 6:** Regression analysis summary predicting avoidance showing emotions.

	Adjusted *R*^2^ = 0.165						
Variables	*B*	*SD*	95% CI	β	*t*	*p*	*VIF*
Age	2.39	0.95	[0.529, 4.256]	0.136	2.56	0.012	1.029
Positive affections	0.460	0.069	[0.324, 0.596]	0.357	6.66	0.000	1.019
Negative affections	–0.237	0.75	[−0.386, −0.089]	−0.172	−3.14	0.002	1.056
Gender	–0.188	1.030	[−2.215, 1.839]	−0.010	−0.18	0.856	1.020

There was an interaction between age, the number of negative and positive affections when predicting showing emotions.

## Discussion

The emotional aspect related to age has not been sufficiently and widely studied. The experience and minimization of negative emotional reactions in the time of COVID-19 may have differences, specially by age. Indeed, how individuals respond emotionally may be an important piece of the puzzle regarding age differences in the links between emotional expressiveness and frequency of negative or positive affection.

This study found age differences in the emotional expressiveness and frequency of negative affections. Regarding the emotional expressiveness, people over 30 years old express less avoidance of their emotions than those below 30 years old. At the same time, they seem to be able to express themselves. These results provide evidence that emotional health and regulation can improve with age (Burr et al., 2020), which teaches that not only physical but mental well-being can be maintained during the pandemic.

In this study, older people appear to be more likely to express their emotions, perhaps as a way to control negative emotional reactions during the COVID-19 pandemic, since some of them correspond to the most vulnerable population with the highest number of risk factors, which can aggravate the prognosis of the disease. As a result, coping with stress would be a mostly perceptual process, from which strategies are generated according to individual needs in a variety of situations that the person may be experiencing (Lazarus and Folkman, 1986; Walker et al., 2004). Furthermore, according to positive existential psychology (Wong, 2009, 2011), this finding reflect how COVID-19 can be an opportunity to perceive that life implies constantly fighting in a world full of obstacles, where the only way to survive is to transform those variables seen as weaknesses and turn them into an advantage for personal growth.

People over 30 years old reported more negative emotion and more positive emotion in their current lives. This finding is not similar to those studies conducted before the pandemic (Allard et al., 2010; Reed et al., 2014). It seems that the well-being of younger people is more associated with the minimization of negative emotional reactions (Charles et al., 2009). Although there is no evidence of an age improvement in emotion understanding due to increased experience in interpreting emotional cues (Phillips et al., 2002). Future research should examine the negative emotional and behavioral reactions of young people to see if the pandemic is not affecting them emotionally or if its long-term effect will only be seen.

Gender turned out to be a covariate that plays a role in predicting the expression of emotions but not in the avoidance of this emotional expression. This result is consistent with what is stated in the studies consulted (Hofstede, 1983; Matsumoto, 1993), in which gender has a role in understanding emotional expression. However, future studies should continue to deepen the study of this variable to further understand its influence, especially when it is combined with other individual variables.

It should be noticed that the low values of the regression weights obtained in both models question the assumption that they might be really sustained. That is why it is recommended to replicate the study using a larger sample or to review the different instruments measuring the positive and negative affections since the obtained results might be due to the managed conceptualization in the instrument used to measure that variable in this research.

Furthermore, future research should include children and adolescents to know what happens in those age ranges, taking into account that previous research suggests that during these ages, differences in emotional expressivity are more visible (Birditt and Fingerman, 2005; Birditt et al., 2011). This should be considered, especially now that we are facing a pandemic for the first time experienced and we cannot yet measure its effect on the emotional health of children and young people.

Due to the above, the provided empiric evidence in this research is the first step and should be complemented by other studies to expand the studied sample in each age group in the time of COVID-19 pandemic. Nevertheless, the obtained findings allow us to conclude that the emotional variables should be included in any treatment. Which would help to better understand in times of crisis, the interaction of both positive and negative life experiences to achieve optimal well-being and mature happiness.

Several limitations of the study should be considered. The first of these is the cross-sectional design of this study. Future longitudinal or prospective studies in this area could help to elucidate the potential role of age in conjunction with other uncontrolled sociodemographic variables, such as profession, occupation, socioeconomic level. This will help prevent emotional disturbances and post-traumatic stress disorder (PTSD) after COVID-19.

Secondly, this study was based on self-report measures through an online survey and, therefore, the results are subject to possible participant response bias. In third place, the sample size was small, this decreased the statistical power of the statistical study. A larger sample size is needed in future research to include more data to verify the results obtained. Fourth, it is recommended to reproduce the study including younger populations in order to generalize the results found to other sectors of the population. Lastly, it would be advisable to replicate the present studies in international samples in order to generalize the results found, as well as to make comparisons.

Despite its limitations, the findings of this research shed light on the ways in which age and emotional expressiveness are related to mental health in the time of COVID-19. People’s capacity and maintenance of well-being can be improved by paying attention to strategies that promote emotional expressiveness in their coping styles. Similarly, the results reinforce the importance of being resilient, and how this variable could explain that older people face a reality of real physical and emotional risks for them, to turn it rather into a moment of overcoming (Apter, 2020).

In summary, the results obtained not only represent a contribution to the therapeutic work and to the participation of the psychologist in post-COVID-19 clinical treatments, but will also allow to contribute to reinforcing the study of positive existential psychology as a post-pandemic science.

## Data Availability Statement

The datasets generated for this study are available on request to the corresponding author.

## Ethics Statement

The studies involving human participants were reviewed and approved by the Pontificia Universidad Catolica de Chile. The patients/participants provided their written informed consent to participate in this study.

## Author Contributions

MG-T conceived of the presented idea. MG-T and SH-R developed the theory, performed the computations, and verified the analytical methods. MG-T encouraged SH-R to investigate new programming languages and strategies for data analysis and supervised the findings of this work. Both authors discussed the results and contributed to the final manuscript.

## Conflict of Interest

The authors declare that the research was conducted in the absence of any commercial or financial relationships that could be construed as a potential conflict of interest.
